# What the Cell Can Do

**Published:** 1883-01

**Authors:** F. Cohn


### ARTICLE IX.

### *What the Cell Can Do.*

Water, earth salts, and the gases—the raw materials which the plants suck up—are changed within the cells into starch and sugar, gum and woody fibre, albumen and wax, oil and resin, into powerful medicines and deadly poisons. The simplest plant possesses an art which the most skillful chemist has not been able to learn from it. It is true that the chemist can artificially prepare in his laboratory many of the substances which the plant-cell likewise produces; he can convert the starch of the potato into the sugar that gives the wine-grape its sweetness; this, again, he can transform into the fruit-acids which, in connection with the sugar, give the berries their fresh and agreeable taste; he can even produce the flavor of the fruits from the fusel-oil which he obtains by the fermentation of the sugar. He can make the oil of bitter almonds from benzoic and formic acids; he can, with as good art, imitate the pungent taste of the pepper, and the biting one of the mustard-seed, and the narcotic poison which only the nightshade has hitherto prepared for the healing of sore eyes. He can produce from the sap of firs the crystal-needles of the vanilla, for which a Mexican orchid has heretofore been obliged to give up its pods; from the distillation of wood he obtains a smoky fluid, from which he procures salicylic acid, for the production of which the flowers of the meadow-sweet or the bark-tissues of the willow were formerly required; and from this he makes also the ink coloring gallic acid, which formerly only a little wasp knew how to draw out by its sting from the cells of the oak, and the aroma of the wood-ruff. He has made the work of the cells in the madder-root superfluous, for he has fabricated its costly dyes, along with a hundred other splendid pigments, out of tar oil and stone-coal, and is now on the point of taking its works away from the indigo-plant by artificially producing indigo. But a raw material which has at some time been brought forth out of the laboratory of a living plant-cell always lies at the foundation of all these manipulations of the chemist, wonderful as they are. And, notwithstanding the immense progress that modern chemistry has made within the last ten years, its art is still limited at this point: no prospect yet exists that it will be able, artificially, to produce the most important of all the substances that go to build up the bodies of animals and plants, and, to form their living cell-tissues—protoplasms, or the envelope of the plant-cells, the matter of the muscles and nerves.—*Professor Ferdinand Cohn, in Popular Science Monthly for December.*

---

